# Real-world Practical Experience of Angiotensin Receptor-neprilysin Inhibitor in Older Japanese Patients with Chronic Heart Failure

**DOI:** 10.31662/jmaj.2023-0109

**Published:** 2023-09-27

**Authors:** Toshinori Komatsu, Masatoshi Minamisawa, Ayako Okada, Hirohiko Motoki, Toshio Kasai, Koichiro Kuwahara, Uichi Ikeda

**Affiliations:** 1 Department of Cardiology, Shinshu University School of Medicine, Nagano, Japan; 2 Department of Cardiology, Nagano Municipal Hospital, Nagano, Japan

**Keywords:** sacubitril/valsartan, angiotensin receptor blocker, older Japanese patients, chronic heart failure, CONUT score

## Abstract

**Introduction:**

Sacubitril/valsartan, an angiotensin receptor-neprilysin inhibitor (ARNI), is superior to enalapril for chronic heart failure (CHF) with reduced ejection fraction (EF). However, its efficacy and safety in older Japanese patients in clinical practice are poorly understood. We aimed to investigate the efficacy and safety of ARNI compared with angiotensin receptor blocker (ARB) in older patients with CHF in real-world clinical practice. In addition, nutritional status and body composition were investigated as essential indicators of efficacy.

**Methods:**

This retrospective single-center observational study enrolled 55 consecutive older patients (aged ≥75 years) with CHF who received ARNI (n = 27) or ARB (n = 28) therapy between October 2020 and March 2021. Blood samples were collected before (baseline) and 4, 12, and 24 weeks after ARNI or ARB therapy initiation. Furthermore, echocardiography was performed before (baseline) and 24 weeks after ARNI or ARB therapy initiation. The efficacy endpoints were changes in N-terminal pro-B-type natriuretic peptide (NT-proBNP) levels, left ventricular EF, nutritional status, and body composition changes. The controlling nutritional status (CONUT) score and geriatric nutritional risk index were investigated as nutritional status indices. The safety endpoints were symptomatic hypotension, renal function exacerbation, and hyperkalemia in patients who continued ARNI or ARB therapy for >24 weeks without additional nonpharmacological treatment.

**Results:**

There were no significant changes in NT-proBNP levels and estimated glomerular filtration rates; however, there was a significant CONUT score improvement in the ARNI group (least-squares mean difference, −1.0; 95% confidence interval, −1.4 to −0.3; *p* = 0.04). The initial ARNI dose could not be uptitrated in five patients (19%) due to hypotension.

**Conclusions:**

ARNI exhibited significant improvement in the nutritional status in older patients with CHF compared with ARB. However, the ARNI dose should be adjusted according to the patient’s blood pressure.

## Introduction

In Japan, the number of patients aged ≥75 years with chronic heart failure (CHF) is rapidly increasing. Various clinical investigations demonstrated the effectiveness of various pharmacological agents and mechanical supports; however, therapeutic strategies for patients aged ≥75 years are yet to be established. Sacubitril/valsartan, an angiotensin receptor-neprilysin inhibitor (ARNI), has become a standard treatment for heart failure (HF). The prospective comparison of ARNI with ACEI to determine impact on global mortality and morbidity in heart failure (PARADIGM-HF) trial showed that ARNI reduced the risk of cardiovascular death, all-cause mortality, and HF recurrence more than enalapril in patients with HF and reduced ejection fraction (HFrEF) ^(1)^. Similar to the PARADIGM-HF trial, a prospective comparative study of ARNI and enalapril in Japanese patients with HFrEF (the prospective comparison of ARNI with ACE inhibitor to determine the novel beneficial therapeutic value in Japanese HF patients [PARALLEL-HF] trial) demonstrated that ARNI was safe and well tolerated in Japanese patients with CHF ^(2), (3)^.

The prospective comparison of ARNI with ARB global outcomes in HF with preserved ejection fraction (PARAGON-HF) trial showed therapeutic benefits in patients with ejection fraction (EF) below the normal range; however, the therapeutic benefits of ARNI among patients with HF and preserved ejection fraction was not demonstrated ^(4), (5)^.

The mean ages of the patients were 63.8, 72.7, and 67.9 years in the PARADIGM-HF, PARAGON-HF, and PARALLEL-HF trials, respectively. Therefore, the usefulness of ARNI in patients aged ≥75 years with HF is insignificant. In recent years, ARNI has been extensively used to treat older patients with CHF; however, its safety and efficacy still need to be investigated using real-world data. The nutritional status of older patients with CHF affects their body composition, physical function, and prognosis ^(6), (7)^; the nutritional status of older patients with CHF can easily deteriorate, and the nutritional deficiencies in adults aged ≥70 years increase the risk of hospitalization for HF, contribute to skeletal muscle loss, and are associated with decreased quality of life ^(6)^. A subanalysis of the PARADIGM-HF trial revealed that ARNI may be involved in glucose metabolism ^(8)^. Furthermore, it has been reported that ARNI improved nutritional status ^(9)^; therefore, we hypothesized that ARNI therapy could improve nutritional status in older patients compared with angiotensin receptor blocker (ARB) and also conducted a comparative study on the safety and efficacy of ARNI and ARB in patients aged >75 years who received ARNI or ARB therapy for CHF. Nutritional status and body composition, which are vital efficacy indicators, particularly in older patients, were investigated.

## Materials and Methods

### Study design and patient population

This retrospective observational study enrolled 55 consecutive older patients (aged ≥75 years) with CHF from Nagano Municipal Hospital who received ARNI (n = 27) or ARB (n = 28) therapy between October 2020 and March 2021. CHF was diagnosed based on the Framingham Heart Study criteria ^(10)^. Patients with CHF with residual left ventricular dysfunction or HF symptoms, despite guideline-based medical therapy in the outpatient clinic or during hospitalization, were selected for ARNI or ARB therapy at the discretion of the cardiologist in charge. ARNI therapy was changed from ARB, and ARB therapy was either initiated as new or changed from ACEI. In the ARB group, ACEI was converted to ARB because ARNI treatment was being considered for future therapy. Direct conversion of ACEI to ARNI would require a 36-h washout period to lower the risk of angioedema. ACEI was converted to ARB as ARB can be converted to ARNI without a washout period. However, during the study period, ARNI was only approved for up to a 2-week prescription, and patients who had difficulty using ARNI due to hospital visits or financial circumstances were consequently assigned to the ARB group. The new ARB therapy was initiated to treat HF. In principle, the starting dose of ARNI therapy was 100 mg/day, and the titrated dose depended on the patient’s systolic blood pressure (SBP; >90 mmHg). The initiation dose of ARB depended on the patient’s SBP at the start. If a patient had SBP of <90 mmHg, the ARNI or ARB dose was adjusted according to the SBP. This study was approved by the institutional review board of Nagano Municipal Hospital (IRB approval code: No. 0021), and informed consent was obtained from the patients using an opt-out approach. Furthermore, the study was conducted in accordance with the Declaration of Helsinki. Blood samples were collected at 4, 12, and 24 weeks after ARNI or ARB therapy initiation. Baseline blood samples were collected when ARNI was changed from ARB and when ARB was newly started or changed from ACEI. Patients’ demographic data, including age, sex, body mass index (BMI), heart rate, SBP, medical history, and information on baseline treatments, were collected. The collected clinical laboratory data included plasma B-type natriuretic peptide (BNP) concentrations, hemoglobin, N-terminal pro-B-type natriuretic peptide (NT-proBNP) concentrations, albumin, total lymphocyte count, creatinine (Cre), estimated glomerular filtration rate (eGFR), potassium, total cholesterol (TC), and low-density lipoprotein cholesterol (LDL-C). Echocardiographic data were measured for efficacy analysis before and 24 weeks after ARNI therapy initiation. Using the modified Simpson method in the apical four- and two-chamber views, left ventricular EF (LVEF) was calculated.

### Evaluation of nutritional status

In this study, the controlling nutritional status (CONUT) score and geriatric nutritional risk index (GNRI) were used to evaluate nutritional status. The CONUT score was developed for hospitalized patients as a screening tool for nutritional status. The following parameters were used to calculate the score: total lymphocyte count (count/mL), TC level (mg/dL), and serum albumin level (g/dL) ^(11)^. We defined CONUT score of ≥5 as a low nutritional status. Thus, immune defenses, caloric depletion, and protein reserves can be assessed using the CONUT score. The GNRI is a useful tool for predicting the risk of morbidity and mortality in hospitalized older patients ^(12), (13)^. We defined GNRI of <92 as low nutritional status. The GNRI was calculated from serum albumin and BMI as follows ^(14)^:

GNRI = 14.89 × serum albumin (g/dL) + 41.7 × present body weight/[(height)2(m^2^) × 22];

therefore, GNRI = 14.89 × serum albumin (g/dL) + 41.7 × BMI/22

### Measurements using a bioelectrical impedance analyzer

Body fat mass, present body fat, intracellular and extracellular water, extracellular water/total body water, skeletal muscle mass, and basal metabolism were measured using a bioelectrical impedance analyzer (InBody S10, Biospace, Tokyo, Japan); however, this device cannot be used in patients with a pacemaker or implantable cardioverter defibrillator implanted because it applies weak electric current. Thus, measurement could not be performed on one patient with an implanted pacemaker in the ARNI group.

### Endpoints

The efficacy endpoints were changes in NT-proBNP levels, LVEF, nutritional status, and changes in body composition. The CONUT score and GNRI were used to evaluate nutritional status.

The safety endpoints were symptomatic hypotension, renal function exacerbation, and hyperkalemia in patients who continued ARNI or ARB therapy for >24 weeks.

### Definitions

We checked the patients’ medical records and extracted their diagnoses. Disease was classified according to the International Statistical Classification of Diseases and Related Health Problems, 10^th^ revision (ICD-10). The definition of ischemic heart disease is a past percutaneous coronary intervention procedure and/or ischemia evaluation is performed and positive. Peripheral artery disease was defined as claudication by limb artery stenosis and an ankle-brachial index of 0.90. Hypertension was defined as a home blood pressure of 135/85 mmHg, clinical room blood pressure of 140/90 mmHg, or the use of oral antihypertensive drugs. Dyslipidemia was defined as fasting serum triglyceride ≥ 150 mg/dL, HDL-C < 40 mg/dL, or the use of oral antihypertensive drugs. Anemia was defined as hemoglobin of ≤12 g/dL (woman) or ≤13 g/dL (man). Diabetes mellitus (DM) was defined as hemoglobin A1c level of ≥6.5% (National Glycohemoglobin Standardization Program value) or receiving medical treatment for DM. Malignancy and stroke were defined as receiving medical treatments for malignancy/stroke or a history of stroke/malignancy before admission. Chronic kidney disease was defined as eGFR of <60 mL/min/1.73 m^2^. The eGFR was calculated from the serum Cre level, age, weight, and sex using the following formula: eGFR = 194 × Cr1.094 × age − 0.287 (man) or eGFR = 194 × Cr1.094 × age − 0.287 × 0.739 (woman).

### Statistical analysis

Continuous variables were expressed as median with interquartile range (IQR) and categorical variables as frequency and percentage. Clinical data were compared between the two groups using unpaired Student’s *t*-test or Mann-Whitney U test.

The primary comparison was the least-squares mean difference (LSMD) between the ARNI and ARB groups at 24 weeks posttreatment. The p value of <0.05 was considered to indicate statistical significance. All analyses were conducted using the SPSS software version 24.0 (IBM Corp., Armonk, N.Y., USA).

## Results

### Comparison of patient clinical characteristics between the ARNI and ARB groups

**Figure 1.**
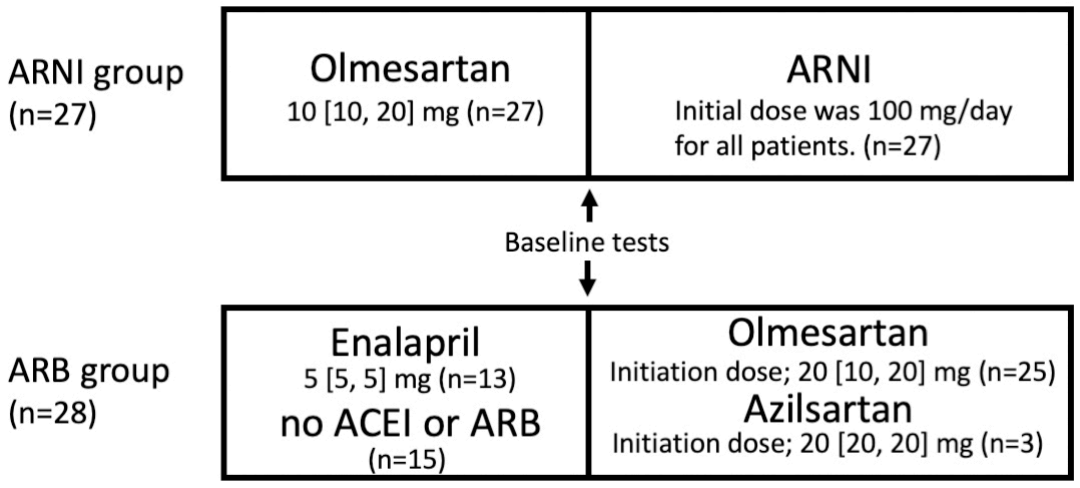
Study scheme. The ARNI and ARB groups enrolled 27 and 28 patients, respectively. In the ARNI group, all patients had been treated with olmesartan prior to ARNI therapy. The initial ARNI dose was 100 mg for all patients and titrated according to blood pressure. In the ARB group, 13 patients used enalapril and 15 patients did not use ACEI or ARB prior to ARB therapy. Olmesartan and azilsartan were used in 25 and 3 patients, respectively. Both olmesartan and azilsartan had a median dose of 20 mg at initiation, and the dose was increased according to blood pressure. ACEI, angiotensin-converting enzyme inhibitor; ARB, angiotensin II receptor blocker; ARNI, angiotensin receptor-neprilysin inhibitor.

A total of 55 consecutive older patients (aged ≥75 years) with CHF who received ARNI (n = 27) or ARB (n = 28) therapy were enrolled in the study. The median follow-up periods of the ARNI and ARB groups were 197 (IQR 180-250) days and 191 (IQR 178-244) days, respectively (*p* = 0.84). The clinical characteristics of the patients are summarized in Table 1. The median ages of the ARNI and ARB groups were 85 and 81 years, respectively (*p* = 0.40). The groups had no significant differences in the baseline median SBP, CONUT score, and GNRI. CONUT score of ≥5 was observed in 13 (48.1%) and 10 (35.7%) patients in the ARNI and ARB groups, respectively (*p* = 0.41). GNRI of <92 was observed in 12 (44.4%) and 9 (32.1%) patients in the ARNI and ARB groups, respectively (*p* = 0.48). There were no significant differences in medical history. Table 1 presents a comparison of the baseline laboratory data and medications between the two groups. There were no significant differences in the baseline median BNP and NT-proBNP concentrations. Diuretics were significantly used in the ARNI group (*p* = 0.04). All patients had been treated with olmesartan prior to ARNI therapy (Figure 1). Furthermore, 13 patients used enalapril, and 15 did not use ACEI or ARB prior to ARB therapy. In the ARB group, olmesartan and azilsartan were used in 25 and 3 patients, respectively, and the dose was increased according to their blood pressure. Olmesartan and azilsartan both had a median dose of 20 mg at initiation. The initial dose of ARNI was 100 mg, which was not increased further in five patients (19%) but increased to 200 and 400 mg in 19 (70%) and 3 (11%) patients, respectively. The median durations required to increase the ARNI dose from 100 mg to 200 and 400 mg were 21 (IQR 15-30) days and 43 (IQR 26-88) days, respectively. The median duration of ARNI administration was 197 (IQR 180-250) days.

**Table 1.** Comparison of Baseline Characteristics, Including Physical Assessment, Nutritional Status, Medical History, Laboratory Data and Medications between the ARNI and ARB Groups.

Characteristic	ARNI (n = 27)	ARB (n = 28)	*p* value
Age (yrs.)	85 [78, 87]	81 [78, 85]	0.40
Female	14 (51.9)	11 (39.3)	0.38
Systolic blood pressure (mmHg)	121 [106, 141]	123 [109, 142]	0.81
Diastolic blood pressure (mmHg)	70 [60, 80]	71 [61, 80]	0.87
Heart rate (beats/min)	69 [58, 83]	73 [61, 82]	0.64
Body-mass index (kg/m^2^)	23.7 [21.8, 26.1]	23.9 [20.8, 26.6]	0.97
CONUT score	4 [2, 5]	3 [2, 5]	0.19
≥ 5	13 (48.1)	10 (35.7)	0.41
GNRI	95.2 [82.7, 99.9]	96.4 [88.7, 102.6]	0.86
<92	12 (44.4)	9 (32.1)	0.48
Medical history
Hypertension	21 (77.8)	20 (71.4)	0.72
Diabetes	8 (29.6)	9 (32.1)	0.85
Dyslipidemia	9 (33.3)	11 (39.3)	0.80
Atrial fibrillation	13 (48.1)	11 (39.3)	0.62
Chronic kidney disease	11 (40.7)	11 (39.3)	0.90
Anemia	8 (29.6)	5 (17.9)	0.38
Stroke	7 (25.9)	5 (17.9)	0.65
Hospitalization for heart failure	27 (100.0)	28 (100.0)	1.00
Ischemic heart disease	5 (18.5)	4 (14.3)	0.77
Peripheral vascular disorder	2 (7.4)	2 (7.1)	0.91
Malignancy	2 (7.4)	4 (14.3)	0.67
Laboratory data
BNP (pg/mL)	330.0 [135.0, 543.2]	398.2 [152.3, 761.0]	0.49
NT-pro BNP (pg/mL)	2100 [1329, 2454]	1605 [1129, 2526]	0.51
Hemoglobin (g/dL)	12.3 [11.3, 12.7]	12.5 [11.5, 13.3]	0.19
Total lymphocyte count (/μL)	1340 [1110, 1780]	1655 [1503, 1810]	0.09
Albumin (g/dL)	3.2 [2.8, 3.9]	3.4 [3.1, 3.7]	0.97
Creatinine (mg/dL)	1.37 [0.80, 1.53]	1.22 [0.89, 1.52]	0.83
eGFR (mL/min/1.73m^2^)	35.8 [28.2, 53.8]	41.1 [28.9, 60.0]	0.43
Potassium (mEq/L)	4.5 [4.4, 4.7]	4.5 [4.1, 4.7]	0.68
Total cholesterol (mg/dL)	151 [138, 191]	155 [139, 197]	0.74
LDL cholesterol (mg/dL)	83 [63, 106]	89 [71, 111]	0.69
Medication
Beta-blocker	17 (63.0)	21 (75.0)	0.47
Mineralocorticoid receptor antagonist	19 (70.4)	19 (67.9)	0.85
SGLT2 inhibitor	3 (11.2)	5 (17.9)	0.60
Ivabradine	2 (7.4)	2 (7.1)	0.87
Tolvaptan	13 (48.1)	8 (28.6)	0.65
Loop diuretic	27 (100)	22 (78.6)	0.04
Statin	14 (51.9)	13 (46.4)	0.68

*P*<0.05 versus ARB by Mann-Whitney U test. Data are presented as median [25%, 75%] or n (%). ARNI, angiotensin receptor-neprilysin inhibitor; ARB, angiotensin II receptor blocker; CONUT, Controlling Nutritional Status; GNRI, Geriatric Nutritional Risk Index. BNP, plasma B-type natriuretic peptide; NT-proBNP, N-terminal pro-B-type natriuretic peptide; eGFR, estimated glomerular filtration rate; SGLT2, sodium-glucose transporter 2; ACE, angiotensin-converting enzyme.

### Efficacy analysis

**Figure 2.**
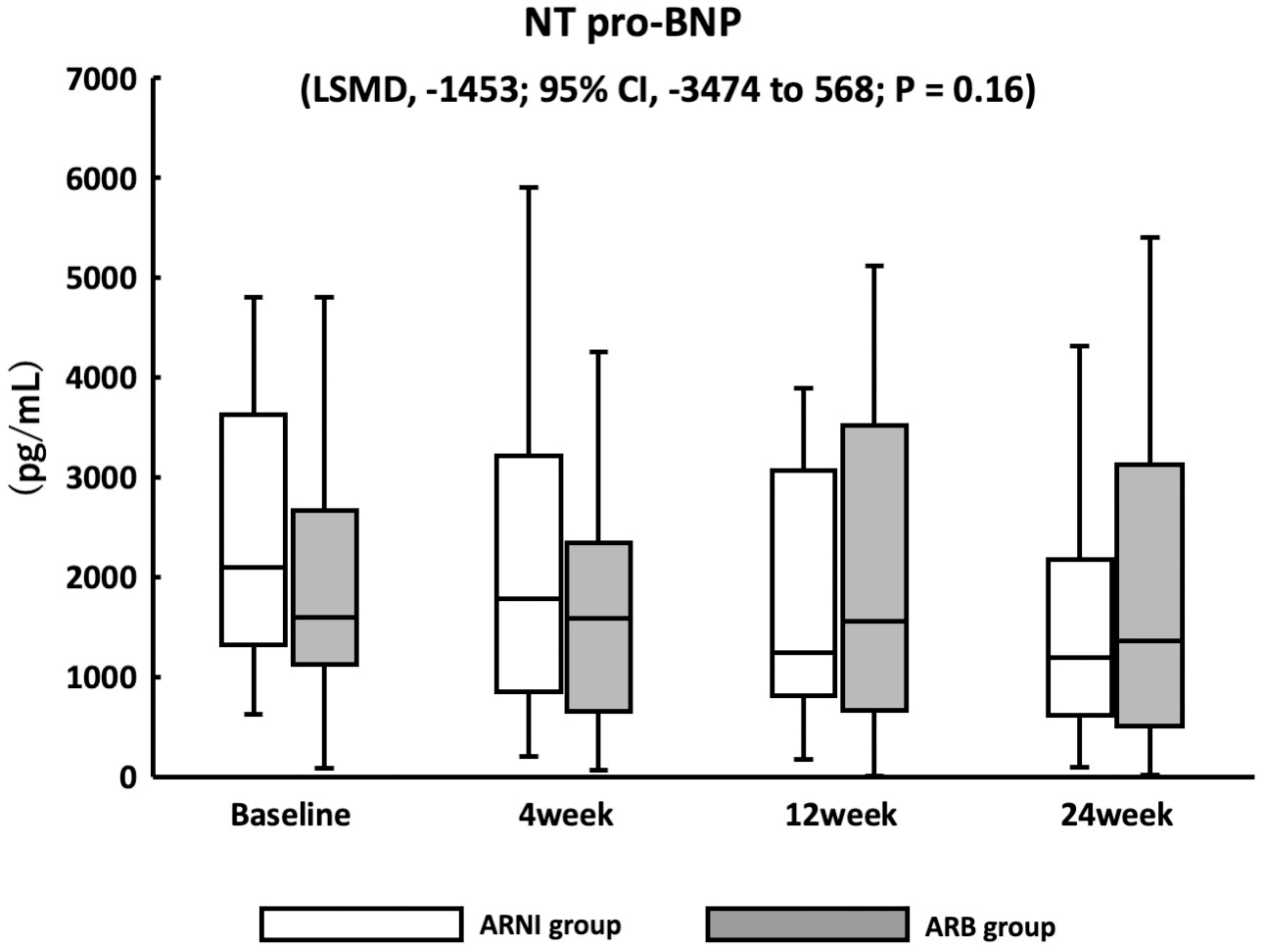
Efficacy analysis based on N-terminal pro B-type natriuretic peptide. There was no significant change in the NT-proBNP levels. NT-proBNP, N-terminal pro B-type natriuretic peptide.

There were no significant changes in NT-proBNP concentrations between the ARNI and ARB groups (LSMD, −1453; confidence interval [CI], −3474 to 568; *p* = 0.16) (Figure 2). Echocardiography was performed at baseline and after 24 weeks of follow-up, and there were no significant changes in all parameters in both groups (Table 2). The baseline EF was significantly lower in the ARNI than in the ARB group (*p* = 0.04). The numbers of patients with HFrEF in the ARNI and ARB groups were 11 (40.7%) and 6 (21.4%), respectively. There was a significant reduction in patients with LVEF of <40% in the ARNI group compared with baseline at 24 weeks post-ARNI therapy (*p* = 0.04).

**Table 2.** Change in the Echocardiographic Parameter between the Baseline and at 24 Weeks after ARNI or ARB Therapy.

	ARNI (n = 27)		ARB (n = 28)		*p*-value			LSMD 95% CI, *p* value
	Baseline	24 weeks	Baseline	24week	Baseline ARNI - ARB	ARNI Baseline - 24week	ARB Baseline - 24week	Baseline-24week ARNI - ARB
LVEDV (mL)	65.5 [53.4, 98.6]	56.0 [48.2, 91.4]	66.4 [54.8, 93.7]	64.3 [54.8, 79.8]	0.71	0.85	0.75	−1.1 (−14.5, 16.6), 0.89
LVESV (mL)	26.1 [15.7, 53.4]	21.3 [14.3, 49.9]	26.1 [15.0, 40.4]	26.9 [15.8, 42.6]	0.82	0.76	0.95	−1.6 (−13.6, 10.4), 0.79
LVEF (%)	56.7 [38.0, 61.2]	61.1 [51.0, 66.3]	60.1. [46.2, 64.4]	60.7 [48.4, 65.9]	0.04	0.22	0.39	1.7 (−2.9, 7.3), 0.47
LVEF >50%	11 (40.7)	17 (63.0)	15 (53.6)	18 (64.3)	0.39	0.48	0.62	-
LVEF 40-49%	5 (18.5)	7 (25.9)	7 (25.0)	6 (21.4)	0.62	0.65	0.76	-
LVEF <40%	11 (40.7)	3 (11.1)	6 (21.4)	4 (14.3)	0.36	0.04	0.56	-
LAV (mL)	65.9 [61.3, 102.0]	61.4 [54.5, 83.2]	64.7 [57.9, 78.3]	66.3 [58.2, 77.1]	0.45	0.31	0.94	−7.1 (−20.0, 5.7), 0.27
LAVI (mL/m^2^)	45.4 [42.1, 62.3]	42.7 [33.7, 53.7]	45.4 [40.8, 54.4]	44.3 [41.2, 54.6]	0.45	0.37	0.91	−4.3 (−14.1, 5.5), 0.39
LAD (mm)	43 [38, 49]	41 [37, 44]	41 [36, 50]	40 [36, 44]	0.68	0.08	0.33	−1.8 (−3.1, 2.3), 0.41
LVDd (mm)	47.0 [42.0, 50.0]	44.5 [39.8, 46.3]	48.5 [41.5, 51.0]	48.5 [40.5, 51.5]	0.32	0.49	0.50	−0.3 (−4.4, 3.8), 0.89
LVDs (mm)	32 [30, 39]	29.0 [26.0, 35.5]	32.0 [28.3, 36.0]	32.5 [26.8, 40.3]	0.44	0.37	0.47	−1.3 (−3.2, 2.8), 0.91
IVST (mm)	11 [10, 12]	11 [10, 12]	12 [10, 12]	11 [10, 12]	0.86	0.99	0.90	−0.1 (−0.9, 1.1), 0.80
PWT (mm)	11 [10, 12]	11 [10, 11]	11 [10, 12]	11 [10, 12]	0.94	0.45	0.30	0.0 (−1.1, 1.1), 0.98
DcT (msec)	194 [151, 208]	208.5 [181.8, 254.5]	195.5 [148.5, 212.0]	215.0 [173.0, 256.5]	0.73	0.32	0.68	2.0 (−35.0, 39.0), 0.92
e' septal (cm/sec)	5.17 [4.80, 6.01]	5.71 [4.70, 7.24]	5.15 [4.24, 6.72]	5.86 [4.25, 6.67]	0.60	0.80	0.58	0.19 (−1.21, 1.59), 0.79
e' lateral (cm/sec)	6.09 [5.10, 8.17]	8.12 [5.38, 9.48]	6.00 [5.09, 8.60]	7.98 [4.76, 9.49]	0.99	0.31	0.90	0.40 (−0.96, 1.76), 0.56
E/e' septal	15.4 [12.6, 22.7]	14.9 [10.8, 22.5]	14.7 [10.2, 19.2]	14.2 [9.8, 19.1]	0.47	0.81	0.72	0.3 (−3.1, 3.6), 0.88
E/e' lateral	13.5 [10.2, 14.8]	12.7 [9.3, 14.4]	11.6 [8.8, 14.8]	11.4 [7.8, 15.7]	0.68	0.82	0.90	0.3 (−2.7, 3.3), 0.83
E/e' mean	14.6 [11.3, 17.6]	14.6 [10.1, 17.7]	14.4 [9.8, 20.9]	13.9 [8.4, 18.0]	0.75	0.91	0.50	1.3 (−1.7, 3.3), 0.39

*p* <0.05 versus ARB and baseline value by Mann-Whitney U test. Data are presented as the median [25%, 75%]. The least-squares mean difference was calculated by analysis of covariance for the change from baseline to 24 weeks. LSMD, least-squares mean difference; CI, confidence interval; ARNI, angiotensin receptor-neprilysin inhibitor; ARB, angiotensin II receptor blocker; LVEDV, left ventricular end-diastolic volume; LVESV, left ventricular end-systolic volume; LVEF, left ventricular ejection fraction; LAV, left atrial volume; LAVI, left atrial volume index; LAD, left atrial diameter; LVDd, left ventricular end-diastolic dimension; LVDs, left ventricular internal dimension in systole; IVST, interventricular septum thickness; PWT, posterior left ventricular wall thickness; DcT, deceleration time; Baseline ARNI - ARB, baseline comparison of ARNI and ARB groups; ARNI Baseline - 24week, Comparison of baseline and 24 weeks after ARNI therapy initiation; ARB Baseline - 24week, Comparison of baseline and 24 weeks after ARB therapy initiation; Baseline-24 week ARNI - ARB, Comparison of ARNI and ARB groups after baseline to 24 weeks after ARNI or ARB therapy.

**Figure 3.**
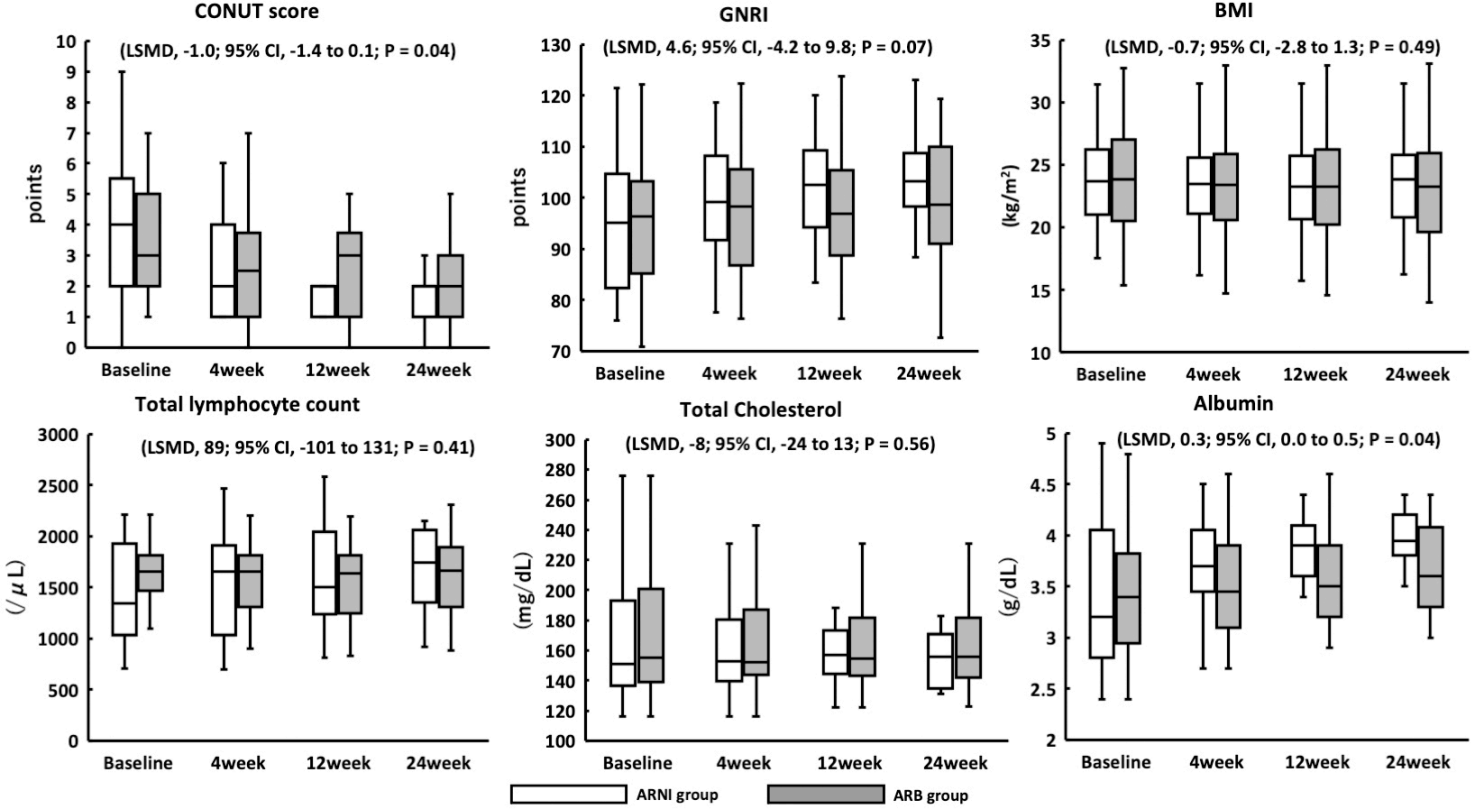
Efficacy analysis based on nutritional status index and related laboratory data. There was a significant decrease in the CONUT score at 4 weeks. There were no significant changes in the GNRI and BMI. Regarding nutritional status, there were no significant changes in the total lymphocyte count and TC; however, the albumin levels significantly increased at 4 weeks. BMI, body mass index; CONUT, controlling nutritional status; GNRI, geriatric nutritional risk index.

In the ARNI group, there was a significant increase in albumin (LSMD, 0.3 g/dL; 95% CI, 0.1 to 0.5; *p* = 0.04); however, there was no significant increase in the total lymphocyte count (LSMD, 89 mg/μL; 95% CI, −101 to 131; *p* = 0.41) and TC (LSMD, −8 mg/dL; 95% CI, −24 to 13; *p* = 0.56) (Figure 3). There was no significant change in BMI (LSMD, −0.7 kg/m^2^; 95% CI, −2.8 to 1.3; *p* = 0.49). In the ARNI group, the CONUT score showed a significant decrease (LSMD, −1.0; 95% CI, −1.4 to −0.3; *p* = 0.04), but there was no significant change in GNRI (LSMD, 4.6; 95% CI, −4.2 to 9.8; *p* = 0.07) (Figure 3). Body composition was measured using a bioelectrical impedance analyzer, and no significant changes were observed for any parameter in either group (Table 3). In the ARNI group, tolvaptan and loop diuretics were discontinued in four and seven patients, respectively, compared with one and two in the ARB group during the study period.

**Table 3.** Change in Body Composition Analyzer between the Baseline and at 24 Weeks after ARNI or ARB Therapy.

	ARNI (n = 26)		ARB (n = 28)		*p*-value			LSMD 95% CI, *p* value
	Baseline	24 weeks	Baseline	24 weeks	Baseline ARNI - ARB	ARNI Baseline - 24week	ARB Baseline - 24week	Baseline-24week ARNI - ARB
Body fat mass (kg)	19.1 [16.4, 24.8]	19.9 [18.2, 26.7]	19.1 [9.8, 25.7]	19.3 [13.3, 25.7]	0.67	0.66	0.46	0.6 (−1.2, 2.1), 0.32
Present body fat (%)	34.5 [30.0, 39.0]	35.4 [30.2, 42.0]	34.1 [21.1, 37.9]	35.4 [22.8, 38.1]	0.32	0.75	0.46	1.6 (−2.4, 3.4), 0.51
Intracellular water (L)	16.1 [14.2, 19.9]	16.0 [14.7, 20.8]	16.7 [15.3, 20.1]	16.9 [14.5, 20.5]	0.34	0.60	0.45	−0.9 (−1.2, 2.0), 0.41
Extracellular water (L)	10.6 [10.1, 19.9]	10.6 [9.9, 12.5]	10.8 [10.3, 13.8]	11.0 [10.3, 13.9]	0.45	0.86	0.41	0.4 (−0.8, 1.6), 0.47
Extracellular water/Total body water	0.41 [0.41, 0.41]	0.41 [0.40, 0.41]	0.40 [0.40, 0.41]	0.41 [0.40, 0.41]	0.28	0.22	0.35	−0.01 (−0.01, 0.00), 0.55
Skeletal muscle mass (kg)	18.7 [16.5, 22.2]	18.0 [16.7, 24.9]	19.7 [18.0, 24.4]	20.0[16.8, 25.5]	0.20	0.89	0.44	−1.7 (−2.0, 2.9), 0.41
Basal metabolism (kcal/day)	1155.5 [1076.3, 1662.3]	1172.0 [1081.5, 1424.3]	1178.5 [1119.0, 1356.0]	1178.5[1094.3, 1380.3]	0.32	0.47	0.89	39.7 (−57.8, 117.2), 0.42

*p* < 0.05 versus ARB and baseline value using the Mann-Whitney U test. Data are presented as the median [25%, 75%]. The least-squares mean difference was calculated by analysis of covariance for the change from baseline to 24 weeks. LSMD, least-squares mean difference; CI, confidence interval; ARNI, angiotensin receptor-neprilysin inhibitor; ARB, angiotensin II receptor blocker; Baseline ARNI - ARB, baseline comparison of ARNI and ARB groups; ARNI Baseline - 24week, Comparison of baseline and 24 weeks after ARNI therapy initiation; ARB Baseline - 24week, Comparison of baseline and 24 weeks after ARB therapy initiation; Baseline-24week ARNI - ARB, Comparison of ARNI and ARB groups after baseline to 24 weeks after ARNI or ARB therapy.

### Safety analysis

**Figure 4.**
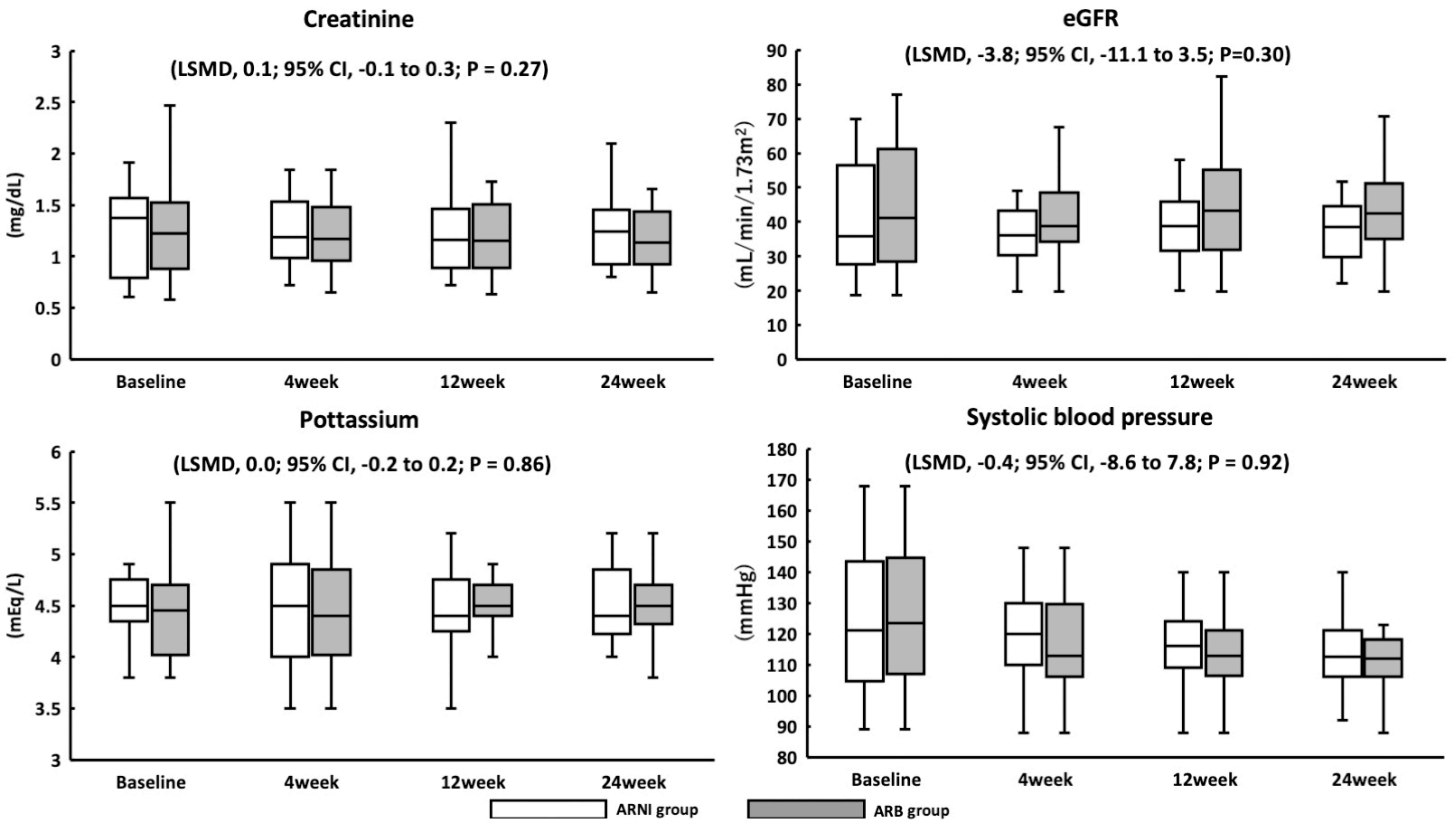
Safety analysis using renal function, potassium, and systolic blood pressure. None of the patients exhibited worsening renal function. No significant increase in potassium levels was observed. Furthermore, there was no significant change in systolic blood pressure after adjusting the ARNI dose. ARNI, angiotensin receptor-neprilysin inhibitor; eGFR, estimated glomerular filtration rate.

This cohort had no mortality from any cause or hospitalization for CHF. Among 27 patients who received ARNI therapy, 7 (26%) had SBP <110 mmHg at initiation, which was an exclusion criterion in the PARAGON-HF trial. Furthermore, two patients (7%) had SBP <100 mmHg at initiation, which was an exclusion criterion in the PARADIGM-HF and PARALLEL-HF trials. Furthermore, five patients (19%) were not titrated from 100 mg, however, none discontinued ARNI therapy within 24 weeks. Among 28 patients who received ARB therapy, 8 (29%) had SBP <110 mmHg, and at the time of initiation, no patient had SBP <100 mmHg. There were no patients with deteriorating renal function or hyperkalemia in the ARNI or ARB group (Figure 4).

## Discussion

In this study, we investigated the efficacy and safety of ARNI therapy by comparing it with ARB in older Japanese patients in real-world clinical practice; there was a significant improvement in the CONUT score in the ARNI group. However, the NT-proBNP did not decrease in the ARNI group compared with the ARB group. SGLT2 inhibitors were recently approved for the treatment of HF during the study period, and prescriptions for older patients were less frequent.

### Efficacy of ARNI therapy compared with ARB therapy

There was no significant improvement in NT-proBNP levels in the ARNI group compared with the ARB group during the study period; however, due to the trend toward improvement, a similar result is expected if the observation period is prolonged. Another factor could be the insufficient number of patients to detect significant changes. This cohort exhibited no significant changes in conventional echocardiographic parameters, including left ventricular systolic and diastolic functions. LVEF of <40% may have contributed to the insignificant difference in LVEF improvement in the ARNI group compared with the ARB group. However, there were significant improvements among patients with LVEF of <40% in the ARNI group, suggesting the possibility of LVEF improvement in older patients as in previous reports ^(1), (2), (3)^.

This study evaluated the representative nutritional status indices, CONUT score, and GNRI as efficacy parameters. It is established that the CONUT score and GNRI are predictors of hospitalization in older patients. The CONUT score significantly improved in the ARNI group compared with the ARB group. The ARNI group showed a 1.0-point improvement in the CONUT score compared with the ARB group in LSMD. A 1.0-point improvement in the CONUT score may contribute to an improvement in all-cause mortality and cardiovascular death. It was hypothesized that nutritional status improvement may play a role in the prognostic effect of ARNI; however, GNRI exhibited no improvement. The CONUT score appears to be influenced by increased albumin levels. The formula for determining the GNRI is significantly dependent on the BMI. We assumed that the lack of change in the GNRI was due to the lack of change in the BMI. Patients with acute HF are often overhydrated, and the BMI often decreases with dehydration. However, the obesity paradox has been proposed, and attention should be given to lowering the BMI of older patients with CHF ^(15)^.

It is well known that a decrease in serum albumin level is correlated with mortality and length of hospital stay due to HF ^(16)^. The European Society for Clinical Nutrition and Metabolism guidelines also indicate the importance of nutritional status, including serum albumin, in patients with CHF ^(17)^. ARNI therapy was previously reported to improve the pre-albumin levels and nutritional status in patients with CHF. It has not been clearly explained that ARNI improves nutritional status, but it has been suggested that enhanced congestion in the gastrointestinal tract improves digestion and absorption. It has also been suggested that the effects of the renin-angiotensin-aldosterone system improve inflammation and reduce albumin consumption ^(18)^. The multifaceted effect of ARNI improves albumin levels, which may be effective for improved prognosis.

A subanalysis of PARADIGM-HF revealed the possibility that ARNI is involved in glucose metabolism, which is presumed to be due to the effect of neprilysin on elevated active GLP-1 concentrations ^(8)^. Such multifaceted effects of ARNI could result in the improvement of nutritional status as reported in previous studies ^(9)^.

It has been suggested that body composition, measured by bioelectrical impedance analysis (BIA), is correlated with sarcopenia ^(19), (20), (21)^. In this study, no significant changes in body composition were observed during the study period, and sarcopenia did not improve. However, measurement with BIA alone is not an accurate assessment, as it should be combined with the measurement of grip strength and dual-energy X-ray absorptiometry.

### Safety of ARNI therapy compared with ARB therapy

In the PARADIGM-HF trial, drug dose reductions and discontinuations as well as symptomatic hypotension were common in patients with low SBP ^(1)^. In the PARALEL-HF study, the most common adverse effect in ARNI was hypotension ^(2)^. The mean SBP in the PARADIGM-HF and PARALLEL-HF studies were 124 and 122 mmHg, respectively. In this study, the ARNI group had a median baseline SBP of 121 mmHg. The ARNI dose could only be increased to 400 mg/day in a few patients, indicating the significance of prescription reviews to prevent lowering blood pressure in older adults. The initiation of ARNI therapy is associated with diuretic dose reduction ^(22)^. No significant deterioration in renal function was observed in this study; this may be due to the ability of several patients to reduce the dose or discontinue the use of diuretics.

### Study limitations

This study had several limitations. First, the sample consisted of patients recruited from a single hospital; furthermore, as it is a retrospective study, it may not be representative of the population. Second, in the evaluation of chronic disease, the treatment period was relatively short. Therefore, a longer study period is needed to investigate the risk and benefits. Third, the type of ARB used for ARB therapy was not standardized. Fourth, baseline EF was significantly different between the ARNI and ARB groups. Fifth, ARNI and ARB were not randomized; thus, confounding factors were not eliminated. Sixth, the CONUT score is a nutritional status index, and whether its improvement indicates actual improvement in nutritional status is unclear. Seventh, no extensively detailed investigation of blood pressure trends was conducted after the initiation of ARNI or ARB therapy. Eighth, changing the therapy from ACEI to ARB is not recommended for the treatment of heart failure, except when intolerance is evident. Finally, these data cannot exclude the effects of concomitantly-administered medications.

### Conclusion

ARNI therapy improved the CONUT score, a nutritional status index, in the treatment of older patients with CHF compared with ARB therapy. In addition, there is a risk of hypotension due to ARNI therapy; thus, the dose of ARNI should be adjusted according to the blood pressure of each patient.

## Article Information

### Conflicts of Interest

None

### Acknowledgement

We thank all the staff of Nagano Municipal Hospital for their cooperation with our research.

### Author Contributions

All authors (1) made substantial contributions to the study concept or the data analysis or interpretation; (2) drafted the manuscript or revised it critically for important intellectual content; (3) approved the final version of the manuscript to be published; and (4) agreed to be accountable for all aspects of the work.

### Approval by Institutional Review Board (IRB)

The IRB approval code is No. 0021 from Nagano Municipal Hospital.
